# Photosystem I cyclic electron flow via chloroplast NADH dehydrogenase-like complex performs a physiological role for photosynthesis at low light

**DOI:** 10.1038/srep13908

**Published:** 2015-09-11

**Authors:** Wataru Yamori, Toshiharu Shikanai, Amane Makino

**Affiliations:** 1 Center for Environment, Health and Field Sciences, Chiba University, 6-2-1 Kashiwa-no-ha, Kashiwa, Chiba 277-0882, Japan; 2 Department of Applied Science, Graduate School of Agricultural Science, Tohoku University, 1-1 Tsutsumidori-Amamiyamachi, Aoba-ku, Sendai, Miyagi 981-8555, Japan; 3 Department of Botany, Graduate School of Science, Kyoto University, Sakyo-ku, Kyoto 606-8502, Japan; 4 PRESTO, JST, 4-1-8 Honcho, Kawaguchi, Saitama 332-0012, Japan; 5 CREST, JST, 4-1-8 Honcho, Kawaguchi, Saitama 332-0012, Japan

## Abstract

Cyclic electron transport around photosystem I (PS I) was discovered more than a half-century ago and two pathways have been identified in angiosperms. Although substantial progress has been made in understanding the structure of the chloroplast NADH dehydrogenase-like (NDH) complex, which mediates one route of the cyclic electron transport pathways, its physiological function is not well understood. Most studies focused on the role of the NDH-dependent PS I cyclic electron transport in alleviation of oxidative damage in strong light. In contrast, here it is shown that impairment of NDH-dependent cyclic electron flow in rice specifically causes a reduction in the electron transport rate through PS I (ETR I) at low light intensity with a concomitant reduction in CO_2_ assimilation rate, plant biomass and importantly, grain production. There was no effect on PS II function at low or high light intensity. We propose a significant physiological function for the chloroplast NDH at low light intensities commonly experienced during the reproductive and ripening stages of rice cultivation that have adverse effects crop yield.

Regulation of photosynthetic electron transport in the thylakoid membrane of chloroplasts is fundamental for the maximum photosynthetic yield and plant growth. The light reactions in photosynthesis convert light energy into chemical energy in the forms of ATP and NADPH. The reactions involve two types of electron flow in the thylakoid membrane. While linear electron transport generates both ATP and NADPH, cyclic electron transport around photosystem I (PS I) is exclusively involved in ATP synthesis without the accumulation of NADPH. ATP and NADPH generated by light reactions are utilized primarily in the Calvin cycle and photorespiratory cycle.

The role of cyclic electron transport around PS I is proposed to be essential for balancing the ATP/NADPH production ratio and/or for protecting both photosystems from the damage via stromal over-reduction^1^. Two pathways of PS I cyclic electron transport have been proposed; the main pathway depends on PGR5 (PROTON GRADIENT REGULATION 5) and PGRL1 (PGR5-LIKE PHOTOSYNTHETIC PHENOTYPE) proteins, whereas the minor pathway is mediated by a chloroplast NADH dehydrogenase-like (NDH) complex^1^. In ArabidoPS Is, the *pgr5* single mutant showed the sensitivity to high light^4^, low CO_2_^5^ and fluctuating light conditions^6^, suggesting that PGR5 plays an important role even for plant growth under the severe field conditions. In rice, the *PGR5* knocked-down lines showed a mild decline in CO_2_ assimilation and biomass production^7^.

On the other hand, NDH-deficient mutants show a sensitivity to various severe stresses, including strong light^8^, low humidity^9^, drought^10^, high temperature^11^, or low temperature^12^, suggesting that NDH might function to alleviate the oxidative stress in chloroplasts^1^. However, the mutant phenotypes are rather mild and the mechanism that chloroplast NDH alleviates oxidative stresses is unclear because of the low rate of electron transport monitored *in vivo* and on isolated thylakoids^13^,^14^. The clear phenotype of NDH-deficient mutants is observed only when the PGR5-PGRL1 protein-dependent pathway is also impaired in the double mutants^15^, indicating that chloroplast NDH may act as a safety valve when the stroma is highly reduced. Although substantial progress has been made in understanding the supercomplex structure of the chloroplast NDH complex with the PS I supercomplex^16^,^17^, the physiological significance of cyclic electron transport around PS I via the chloroplast NDH complex has remained to be clarified.

Abiotic stresses often limit crop productivity and play a major role in determining the geographical distribution of plant species. Thus, understanding the physiological processes underlying stress responses and the mechanisms of tolerance is of immense importance for us. Many studies have focused on the role of PS I cyclic electron transport in alleviation of oxidative damage. However, it has been recently reported that the defect in chloroplast NDH led to a reduction in electron transport at low light intensity in rice^12^ and *Marchantia polymorpha*^18^. Consistently, the size of proton motive force was reduced in the ArabidoPS Is mutants defective in chloroplast NDH at low to moderate light intensities^19^. Because light reactions limit photosynthesis at low light intensity, NDH-dependent PS I cyclic electron transport may play a role in energizing photosynthesis in low light. In this study, the role of NDH-dependent cyclic electron transport in photosynthesis and plant growth was studied in rice under both high and low light. We propose that cyclic electron transport around PS I via chloroplast NDH functions in efficient electron transport at low light intensity in rice.

## Results

### Rice *crr6* mutant do not accumulate the NDH complex

**Figure 1:**
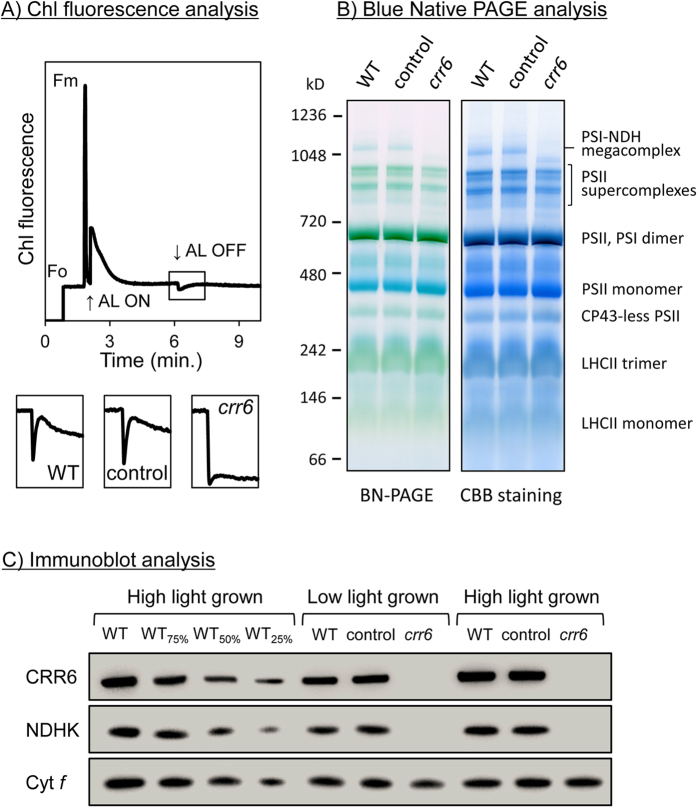
Characterization of the *crr6* mutant by Chl fluorescence analysis and immunoblot analysis. (**A**) Monitoring of NDH activity by chlorophyll (Chl) fluorescence analysis. The curve shows a typical trace of Chl fluorescence in WT plants. Leaves were exposed to actinic light (AL: 200 μmol photons m^–2^ s^–1^) after the measuring light was turned on (F_o_ level). The AL was turned off and the subsequent change in Chl fluorescence level was monitored as an indicator of NDH activity. Leaves were dark -adapted for at least 10 min before the fluorescence analysis. Insets are magnified traces from the boxed area. The fluorescence levels were normalized by the F_m_ levels. NDH activity was monitored by chlorophyll fluorescence in WT plants, plants with the homozygous Tos17 insertion allele (*crr6*; –/–) and plants with the homozygous WT *CRR6* allele (control; +/+). (**B**) Thylakoid protein complexes isolated from WT plants, the control plants and the *crr6* mutant were separated by BN-PAGE (left) and stained with Coomassie Brilliant Blue (CBB) (right). Equal amounts of chlorophyll were loaded per lane. (**C**) Immunoblot analysis of the *crr6* mutant. The leaf extract were separated by SDS-PAGE and immunodetected with specified antibodies. The extracted proteins were loaded on an equal fresh weight basis; the series of dilutions in WT plants is indicated. CRR6 is a stromal protein required for the accumulation of NDH subcomplex A, while NdhK is a subunit of subcomplex A and cytochrome (Cyt) *f* is a subunit of the Cyt *b*_*6*_*/f* complex.

CRR6 is specifically required for the assembly of NdhI in subcomplex A of chloroplast NDH^20^ and was purified in the assembly intermediates of subcomplex A in ArabidoPS Is^21^. The knockout of the *crr6* gene in rice also led to the lack of intact chloroplast NDH^12^. From a progeny of the heterozygous *crr6* plant provided by the National Institute of Agrobiological Sciences of Japan, plants with the homozygous Tos17 insertion allele (*crr6* mutant; –/–) and plants with the homozygous wild-type (WT) *CRR6* allele (control plants; +/+) were identified by PCR analysis and used in the present experiment. Immunoblot analysis showed that the *crr6* mutant did not accumulate the CRR6 protein, resulting in the absence NdhK, a subunit of subcomplex A of the chloroplast NDH complex (Fig. 1), consistent with the previous study^12^. To confirm that the *crr6* mutant had no NDH activity, it was monitored as a transient post-illumination increase in chlorophyll fluorescence, as a result of an NDH-dependent reduction of the plastoquinone pool in darkness^22^. The transient increase in chlorophyll fluorescence was detected in the control and WT plants, whereas it was not observed in the *crr6* mutant (Fig. 1A). The NDH-PS I supercomplex was detected by blue native (BN)-PAGE as a high molecular weight green band in the control and WT plants, but it was not detected in the *crr6* mutant (Fig. 1B). Consistent with the immunoblot analysis, NDH activity was absent in the *crr6* mutant (Fig. 1C).

### Effect of NDH-dependent cyclic electron transport on the plant growth

**Figure 2:**
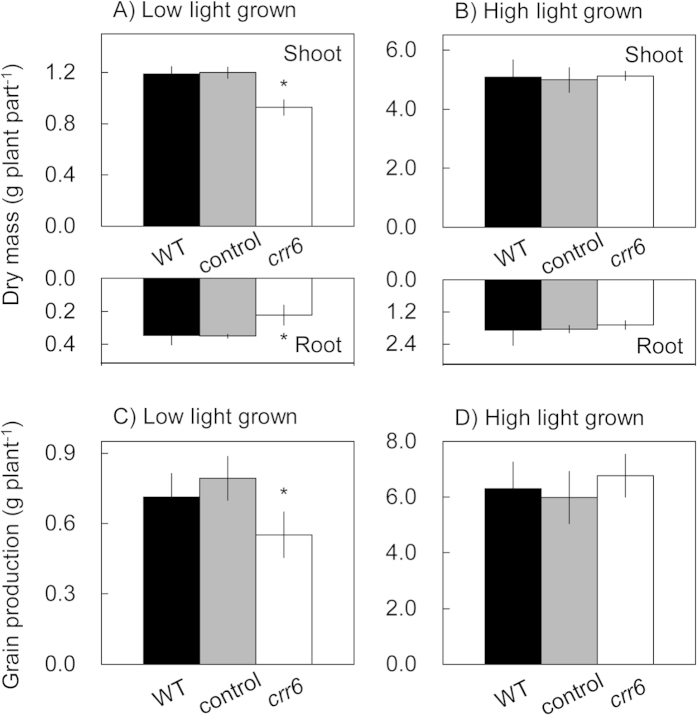
Effect of the *crr6* defect on plant biomass production. Effects of growth light intensity on shoot and root dry weight for 63 days after germination (**A,B**) and on grain production (**C,D**) were analyzed in plants grown at two different growth light intensities. Data represent means ± SE, n = 5 ~ 10. Significant differences among wild type plants, the control plants and the *crr6* mutant are examined by Tukey-Kramer multiple comparison test (*P *< 0.05). When there is a significant difference only in the *crr6* plants compared to the control plants and WT plants, * is indicated.

When rice plants were grown at high light intensity, plant growth and grain production were similar between the control plants and *crr6* mutant (Fig. 2B,D). At low light intensity, however, the *crr6* defect caused reductions in the dry weight of both shoots and roots (Fig. 2A). The grain production in the *crr6* mutant was also decreased at low light intensity (Fig. 2C).

To confirm the genetic link between the Tos17 insertion into the *CRR6* locus and the reduced plant growth at low light intensity, the genotypes of individual F2 plants were determined by PCR (Fig. S1). Plants with the homozygous WT *CRR6* allele (control plants, +/+) and plants with the heterozygous Tos17 insertion (+/–) showed the similar dry weight to the WT plants. In contrast, F2 plants with the homozygous Tos17 insertion (–/–) showed the significant reduction in dry weight than the other genotypes (Tukey-Kramer method, *P *< 0.05; Fig. S1). This result indicates that the growth phenotype at low light intensity genetically links to the *crr6* mutant locus, which results in the absence of NDH in the thylakoid membrane.

### Photosynthetic components in the *crr6* mutant

Growth light had a significant effect on photosynthetic components in WT plants (Table 1), as has been reported^23^,^24^. Contents of leaf nitrogen, Rubisco and cytochrome *f* per unit leaf area were greater in WT plants grown at high light intensity than those of plants grown at low light intensity. In WT plants grown at high light intensity, the chlorophyll contents was higher but the chlorophyll *a*/*b* ratio was lower than those in WT plants grown at low light intensity, respectively (Table 1).

The effect of the *crr6* mutation on the photosynthetic components was also examined under the two different growth light conditions (Table 1). When plants were grown at high light intensity, all the photosynthetic components were similar between the control plants and *crr6* mutant. At low light intensity, however, contents of leaf nitrogen, Rubisco and cytochrome *f* were significantly lower in the *crr6* mutant than in the control plants. In addition, leaf mass per area (LMA) was also lower in the *crr6* mutant than in the control plants, when plants were grown at low light.

### Effect of NDH-dependent cyclic electron transport on the regulation of photosynthesis

**Figure 3:**
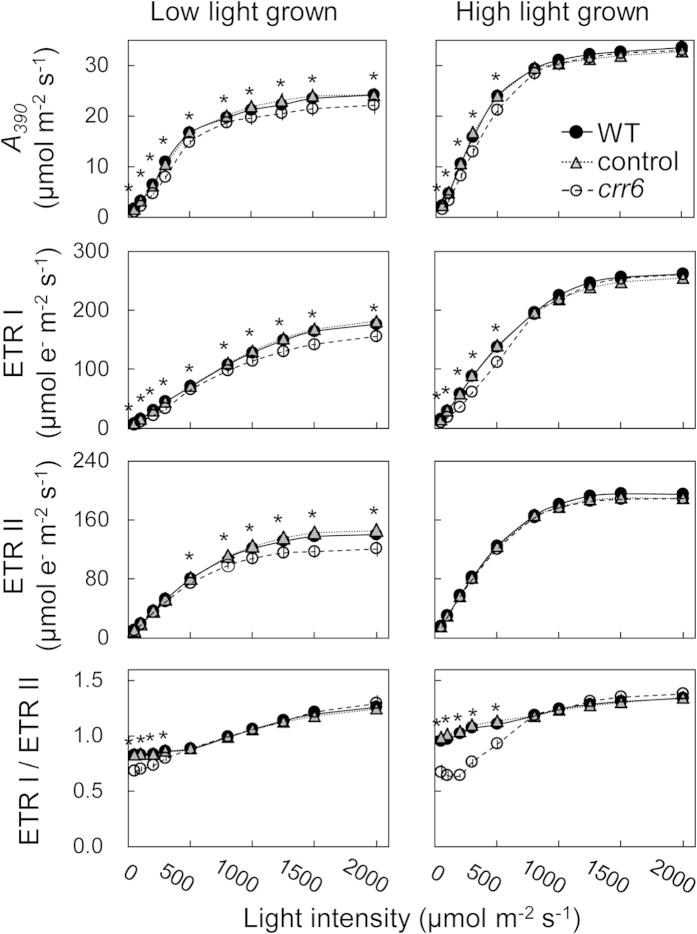
Effect of the *crr6* defect on *in vivo* photosynthesis in plants grown at two different growth light conditions. Light-intensity response of chlorophyll fluorescence, P700 redox state and gas-exchange was simultaneously determined. The electron transport rate at photosystem I (ETR I), electron transport rate at photosystem II (ETR II), the ETR I/ETR II ratio and CO_2_ assimilation rate (*A*_*390*_) at CO_2_ concentration of 390 μmol mol^−1^ were analyzed, as described in Materials & Methods. Data represent means ± SE, n = 4 ~ 6. Significant differences among WT plants, the control plants and *crr6* mutant are examined by a Tukey-Kramer multiple comparison test (*P *< 0.05). When there is a significant difference only in the *crr6* mutant compared to the control plants and WT plants, * is indicated.

Light-intensity responses of several photosynthetic parameters were measured in plants grown under low and high light (Fig. 3, Fig. S2). The electron transport rate through photosystem I (ETR I), through photosystem II (ETR II) were estimated from ϕ PS I and ϕ PS II, respectively, on the assumption that there are no changes in the accumulating ratio of PS I to PS II or their antenna sizes (see, Materials & Methods). When plants were grown at high light, ETR I, ETR II and CO_2_ assimilation rate at CO_2_ concentration of 390 μmol mol^−1^ (A_390_) above the growth light intensity of 800 μmol photons m^−2^ s^−1^ were all similar between *crr6* mutant and the control plants, whereas ETR I and *A*_*390*_ below 800 μmol photons m^−2^ s^−1^ were significantly lower in *crr6* mutant than in the control plants (Fig. 3). In contrast, ETR II below 800 μmol photons m^−2^ s^−1^ were similar between the control plants and *crr6* mutant. As a result, the ETR I/ETR II ratio below 800 μmol photons m^−2^ s^−1^ was lower in *crr6* mutant than in the control plants. On the other hand, when plants were grown at low light, ETR I, ETR II and *A*_*390*_ at all measurement light intensities were lower in *crr6* mutant than in the control plants (Fig. 3). The ETR I/ETR II ratio below 300 μmol photons m^−2^ s^−1^ was lower in *crr6* mutant than in the control plants, but was similar above 300 μmol photons m^−2^ s^−1^. We do not eliminate the possibility that the changes in the accumulating ratio of PS I and PS II or the antenna size may affect ETR I/ETR II. But notably, ETR I/ETR II was different between the *crr6* mutant and the control plants at low light intensity but it was the same at high light intensity (Fig. 3).

**Figure 4:**
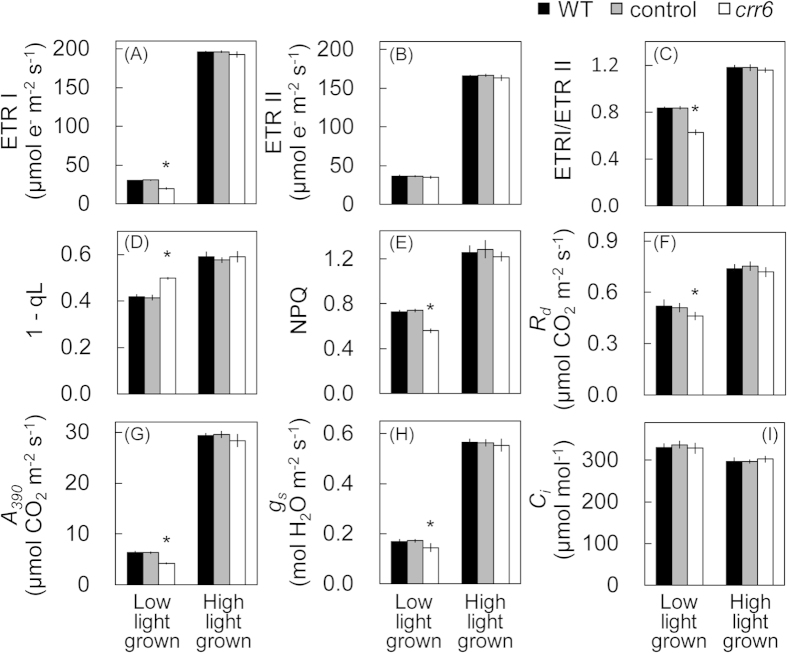
Effect of the *crr6* defect on *in vivo* photosynthesis at the different growth light conditions. Electron transport rate at photosystem I (A; ETR I), electron transport rate at photosystem II (B; ETR II), the ETR I/ETR II ratio (C), the fraction of PS II centers in the closed state (D; 1-qL) and non-photochemical quenching (E; NPQ), dark respiration rate (F; *R*_*d*_), CO_2_ assimilation rate (G; *A*_*390*_), stomatal conductance (H; *g*_*s*_), intercellular CO_2_ concentration (I; *C*_*i*_) at CO_2_ concentration of 390 μmol mol^−1^ at the respective growth light conditions were analyzed, as described in Materials & Methods. The light-intensity response curves of all these parameters are summarized in Fig. 3, S2 & S3. Data represent means ± SE, n = 4 ~ 6. Significant differences among the wild type plants, the control plants and the *crr6* mutant are examined by Tukey-Kramer multiple comparison test (*P *< 0.05). When there is a significant difference only in the *crr6* plants compared to the control plants and WT plants, * is indicated.

Figure 4 summarized several photosynthetic parameters at the same light intensity used for the plant growth. In WT plants, ETR I, ETR II and the ETR I/ETR II ratio at CO_2_ concentration of 390 μmol mol^−1^ were greater at high light intensity than those at low light intensity (Fig. 4). *A*_*390*_, stomatal conductance (*g*_*s*_) and dark respiration rate (*R*_*d*_) were also greater at high light intensity than those at low light intensity (Fig. 4).

The effect of the *crr6* defect on the photosynthetic characteristics was examined at two different light intensities (Fig. 4). When plants were grown at high light intensity, all the photosynthetic parameters including ETR I, ETR II, *A*_*390*_ and *g*_*s*_ were similar between the *crr6* mutant and the control plants (Fig. 4). In contrast, in plants grown at low light intensity, ETR II was similar between the *crr6* mutant and the control plants, but ETR I and also the ETR I/ETR II ratio was lower in the *crr6* mutant than those in the control plants. Reduction in ETR I without the effect on ETR II in the *crr6* mutant resulted in a concomitant reduction of the plastoquinone pool (high 1-qL) and a low transthylakoid pH gradient (ΔpH) (low NPQ) (Fig. 4). The reductions in ETR I likely contributed to the reductions in *A*_*390*_ and *g*_*s*_ (Fig. 4). The *R*_*d*_ was also decreased in the *crr6* mutant than that in the control plants grown at low light intensity.

### Effects of NDH-dependent cyclic electron transport on the alleviation of photoinhibition

**Figure 5:**
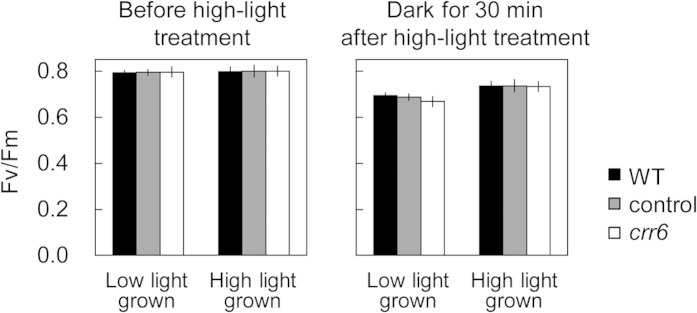
Effect of the *crr6* defect on alleviation of photoinhibition. Effect of the *crr6* defect on photoinhibition was examined. Leaves were exposed to strong light at 2,000 μmol photons m^–2^ s^–1^ at the corresponding temperature for 90 min. The fraction of active PS II (Fv/Fm) was measured after dark incubation for 30 min. Data represent means ± SE, n = 5. Significant differences among wild type plants, the control plants and the *crr6* mutant are examined by Tukey-Kramer multiple comparison test (*P *< 0.05), but no significant differences were observed.

The effect of the *crr6* mutation on the Fv/Fm level was measured after exposure to strong light at 2,000 μmol photons m^−2^ s^−1^ for 90 min in plants grown at two different light intensities (Fig. 5). Before the strong light treatment, Fv/Fm was similar between the *crr6* mutant and the control plants at any growth light intensities. The Fv/Fm level after the light stress was slightly lower in plants grown at low light intensity than those grown at high light intensity, but was similar between the *crr6* mutant and the control plants irrespective of the growth light conditions.

## Discussion

The NDH-dependent cyclic electron transport has been proposed to prevent over-reduction of the stroma under severe stress conditions^1^,^25^. However, it would be true that the NDH-deficient mutants in tobacco and ArabidoPS Is are rather resistant to various stress conditions. On the basis of the low abundance of the complex^26^, and analysis of the rate of electron transport^14^,^27^, NDH-mediated electron flow has been estimated to be too slow to significantly affect ∆pH for ATP production^13^. A question would remain regarding the mechanism how NDH-dependent PS I cyclic electron transport alleviates oxidative stress, if this function in the tolerance is really the case for some stresses.

The present study showed that the impairment of NDH-dependent Cyclic electron transport did not cause any exacerbation of photoinhibition (Fig. 5), any alteration in photosynthetic parameters (i.e., ETR I, ETR II, *A*_*390*_) (Fig. 4), and plant growth (Fig. 2) at high light intensity. On the other hand, at low light intensity, the defect in chloroplast NDH resulted in the reduction in ETR I without any effects on ETR II (Fig. 4), leading to a concomitant reduction in *A*_*390*_ (Fig. 4) and consequently plant biomass and grain production (Fig. 3). Contribution of chloroplast NDH to the steady-state photosynthesis at high light is subtle but we cannot ignore its contribution to photosynthesis and even plant growth at low light in rice. In low light, NDH-dependent cyclic pathway around PS I would participate to the ATP supply and also the tuning of the redox state of intersystem electron carriers, via an additional proton gradient across the thylakoid membrane. This is partly supported by the previous report that substantial up-regulation of the components of PS I reaction center occurs in *ArabidoPS Is thaliana* in response to extremely low light levels during growth^28^. It is also possible that *crr6* disruption could have an effect on the leaf development at low light, resulting in lower photosynthetic capacity (Table 1). In ArabidoPS Is, chloroplast NDH consists of more than 30 subunits and is further associated with PS I to form a supercomplex^16^,^17^. This supercomplex formation could be required for NDH stability, especially under low light, supporting the concept that NDH is machinery for fine-tuning of the chloroplast redox state at low light. Angiosperms may protect chloroplast NDH, a machinery mainly functioning at low light intensity, from oxidative stress at high light intensity by forming the supercomplex with PS I.

We do not eliminate the idea that chloroplast NDH functions as a safety valve when the stroma is highly reduced. This is the case under the *pgr5* mutant background^15^. It has been reported that the photosynthetic activity in the NDH defective rice is somewhat sensitive to the short-term and long-term stress at low temperature^12^. NDH-dependent Cyclic electron transport may prevent the over-reduction of the stroma under the low temperature stress, under which CO_2_ assimilation rate is reduced. Taken together all the information, the NDH complex might have a dual-function role in the photosynthetic regulation as a fine-tuning of the chloroplast redox state at low light and a safety valve that prevents over-reduction of the stroma under various severe stress conditions involving in oxidative stresses. The defect in chloroplast NDH complex accelerates the *pgr5* phenotype, indicating that chloroplast NDH is essential in the absence of PGR5^15^. Further studies are necessary to understand the chloroplast NDH functions at high light intensity by characterizing the phenotype of the double mutant with *pgr5* in rice. The participation of chloroplast NDH to total activity generating proton motive force may be different between rice and ArabidoPS Is.

Rice is a primary food source in the world and mainly grows in a rainy season especially in Asian countries, and is frequently exposed to the shortage in light at various growth stages. In addition, leaves in natural plant canopies experience a highly variable light environment over the course of a day due to changes in leaf angle, as well as the fluctuation in light intensity. Low light (e.g., continuous cloudy days and/or rainfall) during the reproductive and ripening stages has an adverse effect on potential rice yield since the photosynthetic activity is decreased with lowering light intensity^29^. Enhancing photosynthetic capacity of plants is a promising approach to increase crop productivity^30^,^31^. Improving the efficient photosynthesis at low light by through conventional breeding and genetic engineering would be of great importance for producing tolerable cultivars under low light. Our results indicate that NDH-dependent Cyclic electron transport plays an important role for photosynthesis and plant growth at low light intensity and at low temperature. In the northern area of Japan, cool weather damage in summer is always caused not only by low temperature but also by a shortage of sunshine. However, little is known whether plants are potentially able to acclimate to such combined stress conditions of low temperature and low light^32^. Therefore, based on the present discovery, enhancing NDH-dependent Cyclic electron transport could contribute to improvements of photosynthesis and plant growth under such cool weather damage in summer.

## Materials & Methods

### Plant materials and growth conditions

The rice mutant defective in the *OsCRR6* gene (Os08g0167500) by the Tos17 retrotransposon insertion and its wild type (*Oryza sativa* ssp. *japonica* cv. Hitomebore) were used. The plants were grown hydroponically in an environmentally controlled growth chamber as described^12^. The air temperature was 28 °C during a 12-h light period and 23 °C during a 12-h dark period, and the CO_2_ concentration was 390 μmol mol^−1^. Plants were grown under two different light intensities, 200 μmol photons m^−2^ s^−1^ (low light) or 800 μmol photons m^−2^ s^−1^ (high light).

### Analysis of gas exchange, chlorophyll fluorescence and P700 measurements

Measurements of gas exchange, chlorophyll fluorescence and P700 redox state were performed simultaneously with a GFS-3000 and a Dual-PAM-100 measuring system (Walz, Effeltrich, Germany) in uppermost, fully expanded new leaves of 60- to 80-days-old plants as described^12^. After leaves were dark-adapted for 30 min, a saturating pulse was applied to obtain the maximal fluorescence and the maximal change in P700. Several photosynthetic parameters were measured at a CO_2_ concentration of 390 μmol mol^−1^ at various light intensities, after leaves were irradiated for over 30 min to obtain steady-state photosynthesis. The electron transport rate (ETR) was calculated as ETR I (or ETR II) = 0.5 × 0.84 × ϕ PS I (or ϕ PS II), where 0.5 is the fraction of absorbed light reaching PS I or PS II, and 0.84 is the leaf absorptance.

### Analysis of photoinhibition

The leaves were placed in a temperature-controlled chamber at 390 μmol mol^−1^ CO_2_ concentration and 60% relative humidity in a portable gas exchange system (LI-6400, Li-COR, Lincoln, NE, USA). The leaves were exposed to strong light at 2000 μmol photons m^−2^ s^−1^ by a cool light source (PCS-HRX, Nippon Pl, Tokyo, Japan) for 90 min. The fraction of active PS II (Fv/Fm) was measured in the strong-light treated leaves after dark incubation for 30 min with a chlorophyll fluorescence measuring device (Walz, Effeltrich, Germany).

### Quantifications of photosynthetic components and Immunoblot analysis

Immediately after the measurements of gas exchange, leaf samples were taken, immersed in liquid nitrogen and stored at −80 °C. The frozen leaf samples were ground in liquid nitrogen and homogenized in an extraction buffer. Contents of leaf nitrogen, chlorophyll and Rubisco were quantified^12^. Proteins were separated by SDS-PAGE, transferred to a polyvinylidene difluoride membrane. The amount of Rubisco large subunit was determined spectrophotometrically by formamide extraction of the Coomasie Brilliant Blue R-250-stained bands corresponding to the large and small subunits of Rubisco. Contents of CRR6, cytochrome *f* of the cytochrome *b*_6_/*f* complex and NdhK, a subcomplex A subunit of the NDH complex were determined by the immunoblot analysis with each antibody^12^. A dilution series of WT proteins was loaded on gels to estimate the protein level in the mutant. Chlorophyll was extracted in 80% (v/v) acetone and determined^33^. Leaf carbon and nitrogen contents were measured with a CN analyzer (CHNOS Elemental analyzer, Vario EL III, Elementar, Hanau, Germany).

### Thylakoid Membrane Preparation and BN-PAGE Analysis

BN-PAGE was performed as described^20^ with some minor modifications. The freshly isolated thylakoid membranes were gently washed twice with buffer containing 25 mM BisTris-HCl (pH 7.0), 20% glycerol, and solubilized in 25 mM BisTris-HCl (pH 7.0), 20% glycerol, 1.25% DM, at a final chlorophyll concentration of 1 mg ml^−1^. After incubation on ice for 10 min and centrifugation at 12,000 × *g* for 10 min, the supernatants were supplemented with 1/10 volume of BN sample buffer (100 mM BisTris-HCl, pH 7.0, 5% Serva blue G, 0.5 M 6-amino-*n*-caproic acid, 30% sucrose (w/v)). Equal amounts of chlorophyll were loaded in each lane of a gel.

## Additional Information

**How to cite this article**: Yamori, W. *et al.* Photosystem I cyclic electron flow via chloroplast NADH dehydrogenase-like complex performs a physiological role for photosynthesis at low light. *Sci. Rep.* **5**, 13908; doi: 10.1038/srep13908 (2015).

## Supplementary Material

Supplementary Information

## Figures and Tables

**Table 1:** Effect of growth light on leaf properties and photosynthetic components.

Parameter		LMA	Total N	Rubisco	Cyt *f*	Chl	Chl *a/b*
		(g m^−2^)	(mmol m^−2^)	(μmol m^−2^)	(%)	(mmol m^−2^)	
Low light	WT	33.6 ± 0.7a*	99 ± 3a*	3.96 ± 0.21a*	74.1 ± 5.1a*	0.67 ± 0.02a*	3.16 ± 0.03a*
grown	control	34.1 ± 0.5a	95 ± 5a	4.06 ± 0.14a	70.8 ± 3.2a	0.68 ± 0.02a	3.15 ± 0.02a
	*crr6*	30.3 ± 0.9b	84 ± 3b	3.34 ± 0.25b	60.3 ± 4.6b	0.59 ± 0.03b	3.17 ± 0.04a
High light	WT	38.6 ± 0.7a	119 ± 4a	4.81 ± 0.30a	100 ± 3.6a	0.60 ± 0.03a	3.68 ± 0.05a
grown	control	37.8 ± 1.5a	121 ± 5a	4.92 ± 0.35a	94.2 ± 4.7a	0.62 ± 0.05a	3.65 ± 0.06a
	*crr6*	37.4 ± 1.2a	118 ± 4a	5.28 ± 0.42a	95.0 ± 5.0a	0.58 ± 0.04a	3.56 ± 0.05a

Leaf mass per area (LMA), Contents of total nitrogen (Total N), Rubisco, cytochrome *f* (Cyt *f*) and chlorophyll (Chl) were quantified. The Cyt *f* content is shown in a percentage relative to WT plants. Data represent means ± SE, n = 4 ~ 6. Different letters show significant differences in the photosynthetic components among WT plants, control plants and *crr6* mutant at each growth light (Tukey-Kramer multiple comparison test; *P *< 0.05). Asterisks next to WT grown at low light indicate significant differences between data in WT plants grown at low light and high light (Student’s *t* test); *P *< 0.05.
