# Corrigendum: Photosystem I cyclic electron flow via chloroplast NADH dehydrogenase-like complex performs a physiological role for photosynthesis at low light

**DOI:** 10.1038/srep15593

**Published:** 2015-10-29

**Authors:** Wataru Yamori, Toshiharu Shikanai, Amane Makino

Scientific Reports 5: Article number: 13908; 10.1038/srep13908published online: 09112015; updated: 10292015

In this Article, all instances of “ArabidoPS Is” should read “Arabidopsis”

