# Supplementary material for: Photosystem I cyclic electron flow via chloroplast NADH dehydrogenase-like complex performs a physiological role for photosynthesis at low light
Source: Sci Rep. 2015 Sep 11;5:13908. doi: 10.1038/srep13908 (PMC4566099; doi:10.1038/srep13908)

Title

Photosystem I cyclic electron flow via chloroplast NADH dehydrogenase-like complex performs a physiological role for photosynthesis at low light

Authors

Wataru Yamori[1,4], Toshiharu Shikanai[3,5] and Amane Makino[2, 5]

[1]Center for Environment, Health and Field Sciences, Chiba University, 6-2-1 Kashiwa-no-ha, Kashiwa, Chiba, 277-0882, Japan

[2]Department of Applied Science, Graduate School of Agricultural Science, Tohoku University, 1-1 Tsutsumidori-Amamiyamachi, Aoba-ku, Sendai, 981-8555, Japan

[3]Department of Botany, Graduate School of Science, Kyoto University, Sakyo-ku, Kyoto, 606-8502, Japan

[4]PRESTO, JST, 4-1-8 Honcho, Kawaguchi, Saitama 332-0012, Japan

[5]CREST, JST, 4-1-8 Honcho, Kawaguchi, Saitama 332-0012, Japan

Supplemental Figure 1: Cosegregation of the reduced growth phenotype with the *crr6* locus.

Dry mass (g plant^{-1}) at the 63nd day after germination was analyzed in plants grown at low light intensity. Genotypes [homozygous for *Tos17* insertion (–/–), heterozygous for *Tos17* insertion (+/–), the control homozygous (WT*: +/+)] in the progenies of the heterozygous *Tos17* mutant plant and the wild type plants were determined by PCR. The average dry mass (±SD) was 1.54±0.09 g, 1.55±0.08 g, 1.45±0.08 g and 1.13±0.08 g for WT (+/+), WT* (+/+), Hetero (+/–) and Homo (–/–), respectively.

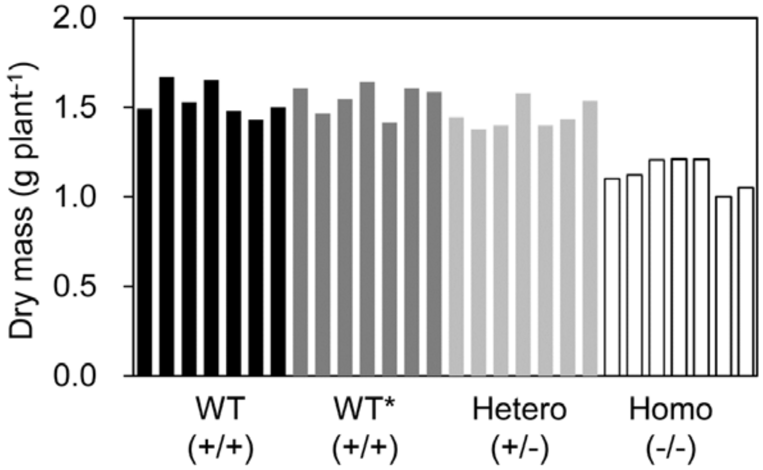

Figure S2: Effect of the *crr6* defect on *in vivo* electron transport activity in plants grown at two different growth light conditions.

Light-intensity response of chlorophyll fluorescence and P700 redox state was simultaneously determined. The electron transport rate at photosystem I (ETR I), electron transport rate at photosystem II (ETR II), non-photochemical quenching (NPQ), and the fraction of PSII centers in the open state (with QA oxidized) (qL) at CO_2 concentration of 390 µmol mol^{-1} were analyzed , as described in Materials & Methods. Data represent means ±SE, n = 4~6. Significant differences among wild-type, the control and *crr6* mutants are examined by a Tukey-Kramer multiple comparison test ($P < 0.05$). When there is a significant difference only in the *crr6* mutant compared to the control plants and wild-type, * is indicated.

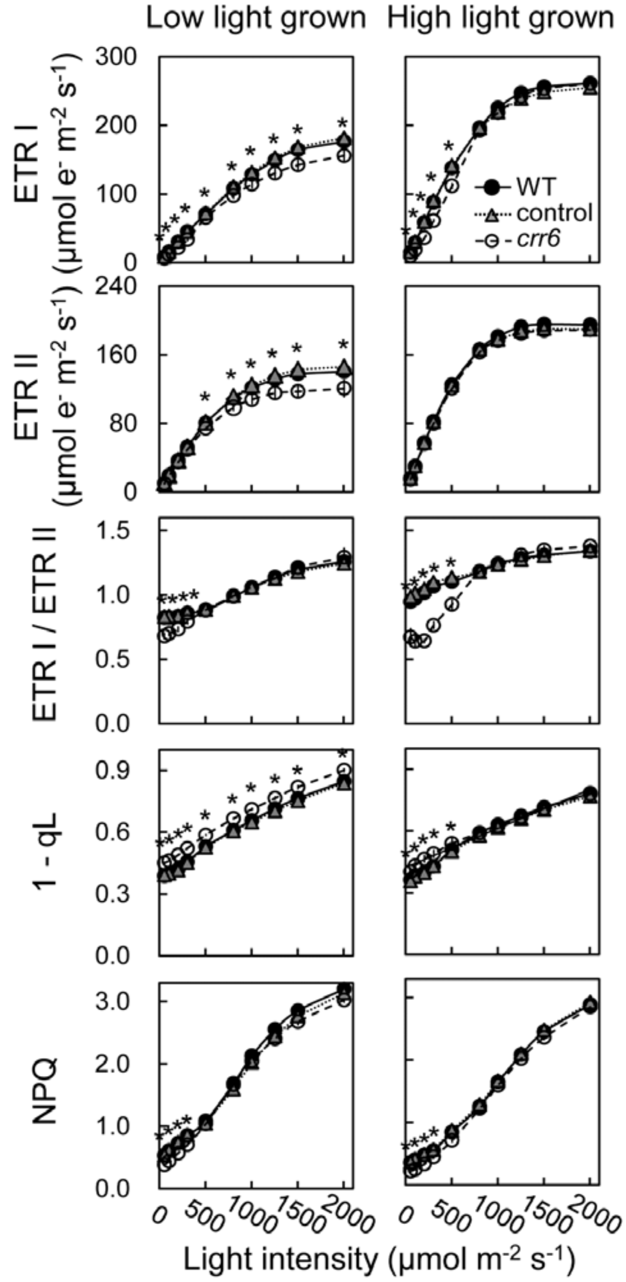

Figure S3: Effect of the *crr6* defect on gas-exchange rate in plants grown at two different growth light conditions.

Light-intensity response of gas exchange rate was determined. The CO_2 assimilation rate (A_{390}), stomatal conductance (g_s), intercellular CO_2 concentration (C_i) and dark respiration rate (R_d) at CO_2 concentration of 390 μmol mol^{-1} were analyzed , as described in Materials & Methods. Data represent means ±SE, n = 4~6. Significant differences among wild-type, the control and *crr6* mutants are examined by Tukey-Kramer multiple comparison test (P < 0.05). When there is a significant difference only in the *crr6* mutant compared to the control plants and wild-type, * is indicated.

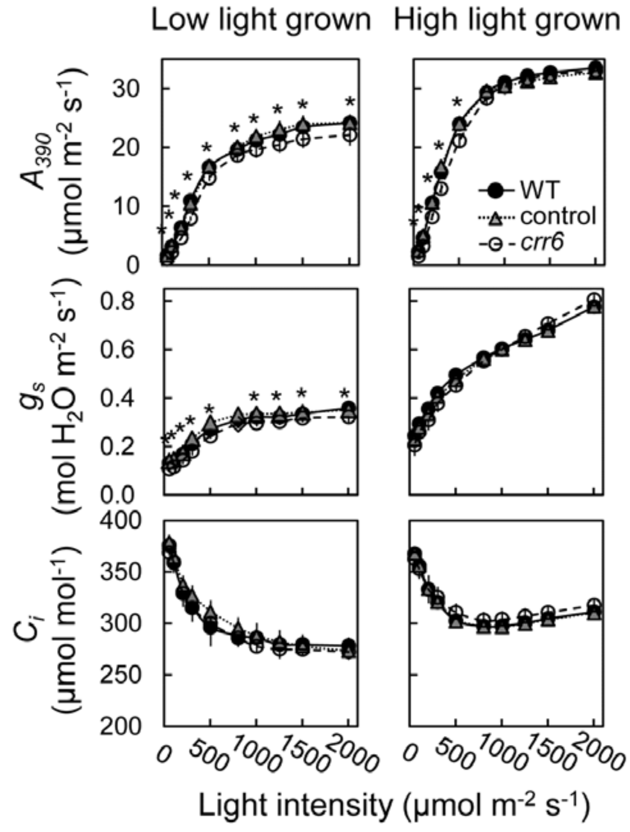

Supplement: Supplementary Information [file srep13908-s1.pdf]
